# Redox-dependent Regulation of Gluconeogenesis by a Novel Mechanism Mediated by a Peroxidatic Cysteine of Peroxiredoxin

**DOI:** 10.1038/srep33536

**Published:** 2016-09-16

**Authors:** Hayato Irokawa, Tsuyoshi Tachibana, Toshihiko Watanabe, Yuka Matsuyama, Hozumi Motohashi, Ayako Ogasawara, Kenta Iwai, Akira Naganuma, Shusuke Kuge

**Affiliations:** 1 Department of Microbiology, Faculty of Pharmaceutical Sciences, Tohoku Medical and Pharmaceutical University, Sendai, Miyagi 981-8558, Japan; 2 Laboratory of Molecular and Biochemical Toxicology, Graduate School of Pharmaceutical Sciences, Tohoku University, Sendai, Miyagi 980-0861, Japan; 3 Department of Medical Biochemistry, Graduate School of Medicine, Tohoku University, Sendai, Miyagi 980-8575, Japan; 4 Department of Gene Expression Regulation, Institute of Development, Aging and Cancer, Tohoku University, Sendai, Miyagi 980-8575, Japan

## Abstract

Peroxiredoxin is an abundant peroxidase, but its non-peroxidase function is also important. In this study, we discovered that Tsa1, a major peroxiredoxin of budding yeast cells, is required for the efficient flux of gluconeogenesis. We found that the suppression of pyruvate kinase (Pyk1) via the interaction with Tsa1 contributes in part to gluconeogenic enhancement. The physical interactions between Pyk1 and Tsa1 were augmented during the shift from glycolysis to gluconeogenesis. Intriguingly, a peroxidatic cysteine in the catalytic center of Tsa1 played an important role in the physical Tsa1-Pyk1 interactions. These interactions are enhanced by exogenous H_2_O_2_ and by endogenous reactive oxygen species, which is increased during gluconeogenesis. Only the peroxidatic cysteine, but no other catalytic cysteine of Tsa1, is required for efficient growth during the metabolic shift to obtain maximum yeast growth (biomass). This Tsa1 function is separable from the peroxidase function as an antioxidant. This is the first report to demonstrate that peroxiredoxin has a novel nonperoxidase function as a redox-dependent target modulator and that pyruvate kinase is modulated via an alternative mechanism.

Peroxiredoxin (Prx) is a member of a family of thioredoxin-dependent peroxidases found in species ranging from *Escherichia coli* to humans^1^,^2^. Prx can reduce H_2_O_2_ using electrons from NADPH in the thioredoxin-dependent redox system of thiol-disulfide exchange between two catalytic cysteines: a peroxidatic cysteine is oxidized to sulfenic acid by H_2_O_2_, and then it reacts with a resolving cysteine of a homodimer counterpart to form a disulfide bond. Enzymatic characterization has indicated that at low H_2_O_2_ concentrations, Prx can efficiently compete with other peroxidases such as catalase and glutathione peroxidase^3^. In fact, a major Prx of yeast *Saccharomyces cerevisiae*, Tsa1, is partially oxidized in unstressed cells^4^ and modulates intracellular reactive oxygen species (ROS) levels^5^. Tsa1 may have a physiological function, even under non-stressed conditions. For example, during stationary growth phase, the antioxidant (peroxiredoxin) function of Tsa1 is associated with life extension^6^, and loss of Tsa1 results in impaired growth during the late exponential and stationary growth phases in rich glucose medium^7^,^8^,^9^. Recently, we reported that lowering ROS levels does not functionally compensate for the loss of Tsa1, leading us to speculate that its role in carbon metabolism is likely due to a non-peroxidase function of Tsa1^5^.

In this report, we describe attempts to define the molecular mechanisms for the alternative functions of Tsa1 that affect metabolic alterations in response to changes in nutrient availability. We identified pyruvate kinase (Pyk1) as a binding protein for Tsa1. Pyruvate kinase (PK) catalyzes an irreversible reaction that generates ATP and pyruvate from phosphoenolpyruvate (PEP) and ADP as the final step in glycolysis (see Fig. 1A for the metabolic map), and it is essential when a fermentable carbon source such as glucose is used^10^. Similarly to the mammalian homologue PK PKM2^11^, a high level of PK activity is maintained during glucose metabolism due to high levels of fructose-1,6-bisphosphate (FBP), an allosteric activator of Pyk1^12^. When glucose is exhausted, ATP synthesis depends on the tricarboxylic acid (TCA) cycle, and cells continue to require glucose-6-phosphate (G6P) to synthesize essential metabolites (see Fig. 1A). The reverse pathway (gluconeogenesis) of G6P synthesis begins with oxaloacetate to form phosphoenolpyruvate (PEP), which is a substrate for Pyk1. Pyk1 is catalytically active, even under conditions of glucose and FBP exhaustion^13^. In fact, Pyk1 plays an important role in the synthesis of several amino acids from pyruvate via gluconeogenesis using ethanol as a carbon source^14^. However, Pyk1 may need to be negatively regulated to avoid energy-wasting cycling reactions and enable efficient gluconeogenesis.

After the transition of glycolysis to gluconeogenesis, gluconeogenic metabolism produces catabolites not only for cell growth but also for the establishment of cell integrity during the stationary phase: the accumulation of trehalose and glycogen are required for desiccation tolerance^15^ and metabolic storage^16^, respectively.

We demonstrate that the peroxidatic cysteine of Tsa1, which participates in its interaction with Pyk1, has a crucial role in achieving an efficient shift from glycolysis to gluconeogenesis. Our results suggest a novel regulatory mechanism for Pyk1 and an alternative non-antioxidant function of Tsa1 (Prx).

## Results

### Effect of Tsa1/2 loss on yeast growth in ethanol medium

**Figure 1:**
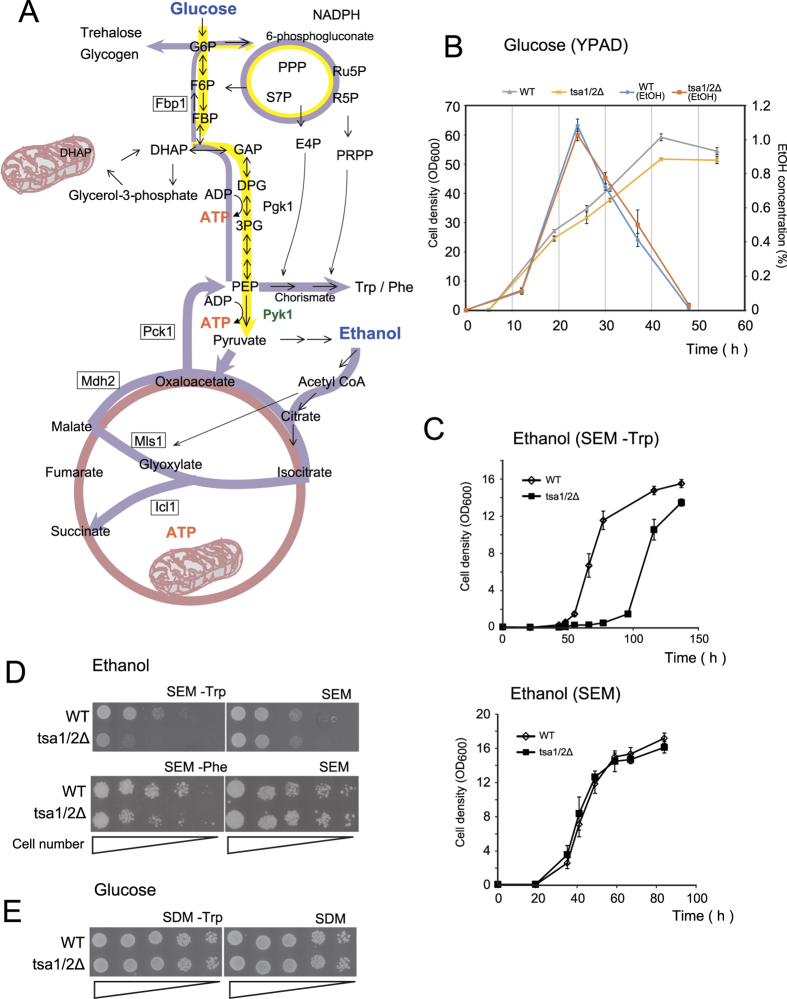
Loss of Tsa1/2 promotes growth suppression in Trp- or Phe-free ethanol medium. (**A**) Schematic representation of glucose (yellow arrows) and ethanol (purple arrows) carbon metabolism. Glycolysis metabolites are abbreviated as follows: glucose 6-phosphate (G6P), fructose 6-phosphate (F6P), fructose 1,6-bisphosphate (FBP), dihydroxyacetone phosphate (DHAP), glyceraldehyde 3-phosphate (GAP), 1,2-diphosphateglycerate (DPG), 3-phosphoglycerate (3PG) and phosphoenolpyruvate (PEP). Metabolites in the pentose phosphate pathway (PPP) include 6-phosphogluconate, ribulose 5-phosphate (Ru5P), ribose 5-phosphate (R5P), sedoheptulose 7-phosphate (S7P), erythrose 4-phosphate (E4P) and phosphoribosyl pyrophosphate (PRPP). Trp/Phe synthesis begins with PEP and E4P via chorismate. Utilization of two-carbon compounds such as ethanol and acetate require the glyoxylate cycle and gluconeogenesis (purple arrows). The glyoxylate cycle plays an anaplerotic role in the provision of metabolites to produce essential compounds in the cytoplasm. The key enzymes of the glyoxylate cycle, isocitrate lyase (Icl1) and malate synthase (Mls1), as well as malate dehydrogenase (Mdh2), are upregulated in yeast in response to the ethanol carbon source and are expressed in the cytoplasm (http://www.yeastgenome.org/). Gluconeogenesis uses most of the enzymes that are common to glycolysis, except during the steps responsible for the synthesis of PEP and F6P, which are catalyzed by phosphoenolpyruvate carboxykinase (Pck1) and fructose 1,6-bisphosphatase (Fbp1), respectively. The tricarboxylic acid TCA cycle is indicated by a bright red-colored circle. (**B**) Growth curves and changes in ethanol levels of wild type (WT, gray and blue lines, respectively) and tsa1/2∆ (yellow and orange lines, respectively) in glucose-rich medium (YPAD). Ethanol was measured using F-Kit Ethanol (J.K. International, Tokyo, Japan). (**C**) Growth curves of wild type (open diamonds) and tsa1/2∆ (closed square) cells, neither of which are Trp/Phe-auxotrophs, growing in Trp-free ethanol medium (SEM –Trp) and Trp-containing ethanol medium (SEM). The data are presented as the mean +/− standard error of the mean (N = 3). (**D,E**) Spot assays were performed on wild type (WT) and tsa1/2Δ cells prepared in SDM culture (early stationary phase, 3 days). A series of one-fifth dilutions of 5 × 10^4^ cells were spotted on agar plates (SEM –Trp, SEM –Phe, SEM, SDM –Trp, and SDM). See also Figure Supplement 1.

In the yeast *S. cerevisiae,* as in many other eukaryotic cells, a high glycolytic flux during exponential growth in glucose results in the production of overflow metabolites, even under aerobic conditions (the Crabtree effect), whereas a low glucose gluconeogenic flux produces assimilation products by consuming overflow metabolites. Ethanol is a significant component of the overflow metabolites of yeast^13^ (see also Fig. 1B). The transition from high to low glucose in the late exponential phase of growth is called the “diauxic shift”^17^, and we reasoned that Tsa1 might affect gluconeogenic flux during such a shift^5^. As shown Fig. 1B, we found that the loss of Tsa1/2 (both *TSA1* and its orthologue *TSA2* were disrupted) did not affect the time-dependent production of ethanol as a metabolite or its consumption. However, as previously indicated^7^,^8^,^9^, growth of tsa1/2Δ cells was retarded at the diauxic shift; thus, assimilation of ethanol might be affected by the loss of Tsa1/2. Therefore, we focused our attention on the effect of the loss of Tsa1/2 on yeast growth using ethanol. We initially compared the growth rate of wild type and Tsa1/2-deficient cells in ethanol medium containing amino acids (SEM) and observed a striking growth defect of tsa1/2Δ cells when Trp was omitted from the medium (Fig. 1C). This was also the case in agar medium without Trp and/or Phe (Fig. 1D; see also Fig. 3B). In contrast, such a growth defect was not observed in glucose medium (SDM –Trp, Fig. 1E).

**Figure 2:**
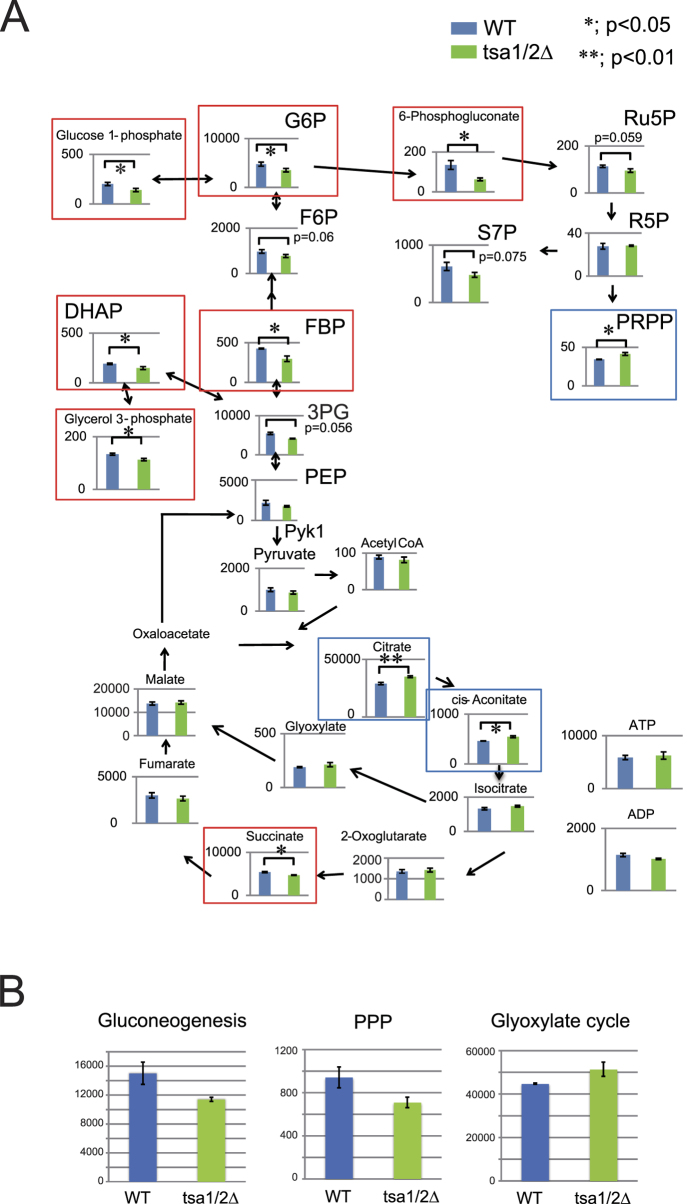
Metabolomic analysis of wild type and tsa1/2∆ cells in SEM –Trp. (**A**) Metabolite levels in wild type (WT, blue bars) and tsa1/2∆ (right green bars) cells exponentially growing in SEM –Trp (OD_600_ = 0.8–0.9) were determined by metabolomic analysis. The data are presented as the mean (pmols/10 OD_600_ units cells) +/− standard error of the mean (N = 3). Red and blue boxes indicate metabolites that were significantly decreased and induced in tsa1/2 cells, respectively. “*” and “**” indicate p values < 0.05 and <0.01, respectively. Some p values greater than 0.05 are also indicated. (**B**) The total amounts of metabolites in gluconeogenesis (PEP, 3PG, DHAP, glycerol 3-phosphate, FBP, F6P, G6P and glucose 1-phosphate; p = 0.069), PPP (6-phosphogluconate, Ru5P, R5P, S7P and PRPP; p = 0.049), and the glyoxylate cycle (citrate, cis-aconitate, isocitrate, glyoxylate and malate; p = 0.021).

Trp and Phe are formed from phosphoenolpyruvate (PEP) in the chorismate pathway. Chorismate is derived from PEP and erythrose-4-phosphate (E4P), which is a metabolite of the pentose phosphate pathway (PPP, Fig. 1A). To gain insight into the metabolic alterations caused by a defect in Tsa1/2, we compared metabolites in wild type and tsa1/2Δ cells grown in SEM –Trp (Fig. 2A,B) and observed in the mutant cells a decrease in metabolites associated with gluconeogenesis (FBP, G6P, glucose-1-phosphate, glycerol-3-phosphate and DHAP) and in PPP (6-phosphogluconate, Ru5P and S7P). In contrast, metabolites of the glyoxylate cycle (citrate, cis-aconitate, and a representative metabolite glyoxylate) were increased, probably as a result of a reduction in PEP. In addition, an increased level of phosphoribosyl pyrophosphate (PRPP) was observed in tsa1/2Δ cells (Fig. 2A). Because both PRPP and chorismate are required for Trp/Phe synthesis (see Fig. 1A), decreased levels of PEP in tsa1/2Δ cells in SEM –Trp could result in Trp/Phe-dependence for efficient growth and explain why the higher levels of PRPP. These results suggest that a defect in Tsa1/2 results in impaired gluconeogenesis, explaining the dependence on Trp/Phe for efficient growth in SEM.

### Pyk1 is a possible regulatory target for Tsa1

**Figure 3:**
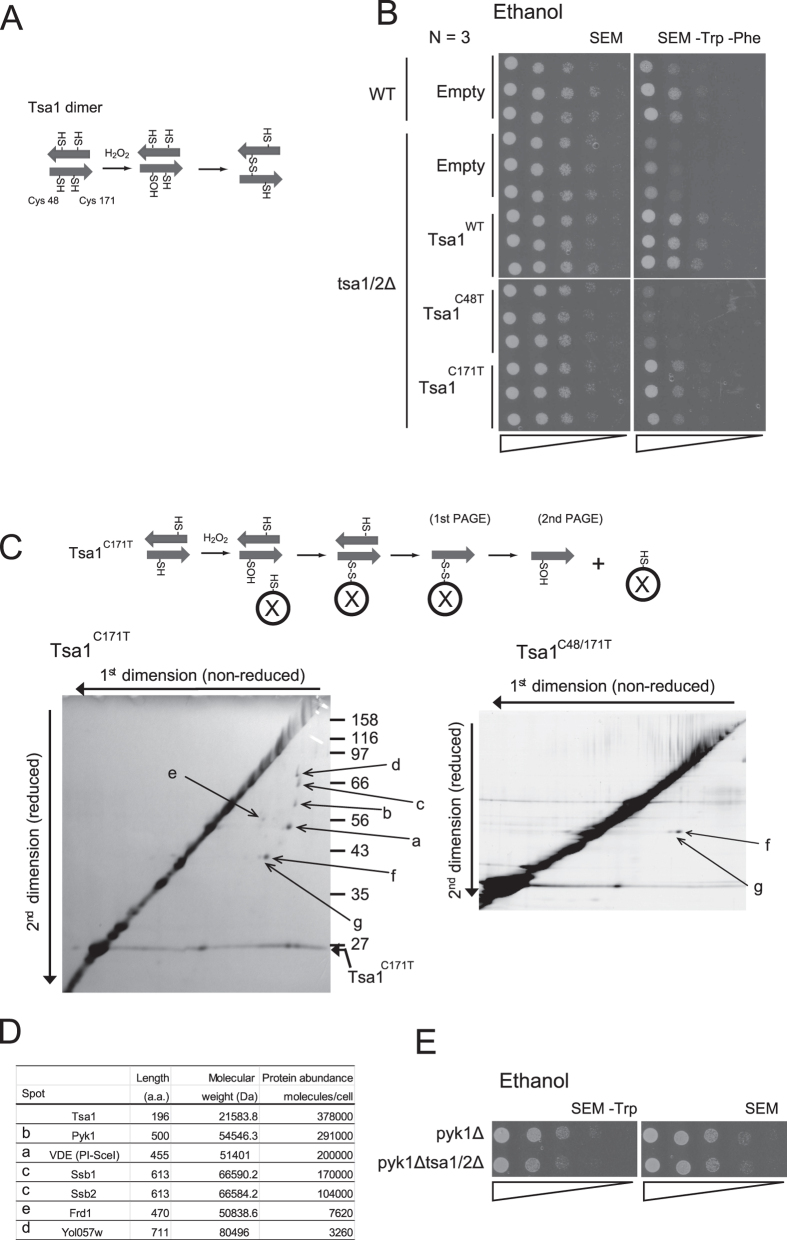
Isolation and identification of Tsa1 binding proteins. (**A**) A schematic illustration of the Tsa1 homodimer. In response to H_2_O_2_, an intermolecular disulfide bond is formed between an H_2_O_2_-induced sulfenic acid from a peroxidatic Cys48 and a resolving Cys171 of the partner (disulfide dimer). (**B**) Spot assay for tsa1/2∆ cells carrying plasmids (pRS315) for Tsa1^WT^, Tsa1^C171T^, and Tsa1^C48T^ on SEM or SEM –Trp –Phe (N = 3). ‘Empty’ indicates cells carrying empty vector, pRS315. For plasmid selection, Leu was also omitted from the medium. ‘WT’ indicates the parental strain BY4742 control cells expressing endogenous Tsa1. (**C**) H_2_O_2_-induced sulfenic acid formation in Cys48 (Cys48-OH) is thought to be stabilized in Tsa1^C171T^. Thus, putative target proteins (X) that can react with Cys48-SOH may be trapped using HAT-tagged Tsa1^C171T^. Tsa1^C171T^ binding proteins were purified from yeast cells (BY4742 *tsa1*Δ) expressing HAT-tagged Tsa1^C171T^. Cells were grown in SDM –Leu and were treated with 0.5 mM H_2_O_2_ for 5 min. Proteins in the purified fraction were fractionated by two-dimensional gel electrophoresis. The first dimension (horizontal) and the second dimension (vertical) were performed under non-reduced and reduced conditions, respectively (left panel). Non-specific proteins that interacted with HAT-tagged Tsa1^C48T, C171T^ mutants were also fractionated (right panel). A gradient gel (10% to 16%) was used for the second dimension. (**D**) Amino acid length, molecular weight (http://www.yeastgenome.org/), and protein abundance^32^ for each spot are shown. See also Figure Supplement 2 for the amino acid sequence information. (**E**) Spot assay of pyk1∆ cells and pyk1∆ tsa1/2∆ cells on SEM –Trp agar plates. Representative data are reported, and the original data (N = 3) are shown in Figure Supplement 1.

Typical Prx enzymes such as Tsa1 have two catalytic cysteine residues (Fig. 3A): a peroxidatic cysteine (Cys48) which reacts directly with H_2_O_2_ to form a Cys-sulfenic acid, followed by the formation of a disulfide bond with a resolving cysteine (Cys171) in a homodimer partner molecule^1^. Both cysteines are essential for the peroxidase activity of Tsa1. We addressed whether these Tsa1 cysteines are required for efficient growth in SEM –Trp –Phe. As shown in Fig. 3B, growth was rescued by the wild type (Tsa1^WT^) and the C171T mutant (Tsa1^C171T^) of Tsa1, but not by the C48T mutant (Tsa1^C48T^), showing that although Tsa1-Cys48 has a crucial role in the growth phenotype, the peroxidase activity of Tsa1 is dispensable.

We have previously demonstrated that Cys48 of Tsa1 acts as a peroxide receptor, inducing a transient interaction with the transcription factor Yap1 via a disulfide bond, and that this interaction is enhanced by a resolving cysteine mutation (Tsa1^C171T^)^18^. We sought other putative Tsa1 target proteins that could link to Cys48 of Tsa1^C171T^ via a disulfide bond using unreduced (1^st^) vs. reduced (2^nd^) two-dimensional SDS-polyacrylamide gel electrophoresis (SDS-PAGE) to separate proteins from immunoprecipitates of HA-tagged Tsa1^C171T^ (Fig. 3C). We performed peptide mass fingerprinting using MALDI-TOF MS and identified five different proteins (Fig. 3C,D): PI SceI (intein Vde1), Pyk1 (also known as Cdc19; pyruvate kinase), Ssb1/Ssb2 (cytosolic HSC70/HSP70 family), Yol057w (dipeptidyl-peptidase III), and Frd1 (soluble fumarate reductase) (see Figure Supplement 2 for amino acid sequence). The failure to identify Yap1 as a binding partner of Tsa1 in this assay may be due to its low abundance^18^. Pyk1, which present in the cell at a similar levels to Tsa1 (Fig. 3D), plays an important role in the rate-limiting step of glycolysis^13^. Although the PK activity of Pyk1 is repressed in the absence of the allosteric activator FBP, residual PK activity may need to be repressed to accomplish efficient gluconeogenesis because gluconeogenesis begins with PEP, a substrate of Pyk1 (see Fig. 1A). To examine the genetic interactions of Pyk1 with Tsa1, we measured the effect of *PYK1* disruption on the growth retardation of tsa1/2Δ cells in SEM –Trp. As shown in Fig. 3E, disruption of *PYK1* resulted in complete suppression of the growth phenotype.

### Downregulation of PK activity by an enhanced interaction between Tsa1 and Pyk1 during gluconeogenesis

**Figure 4:**
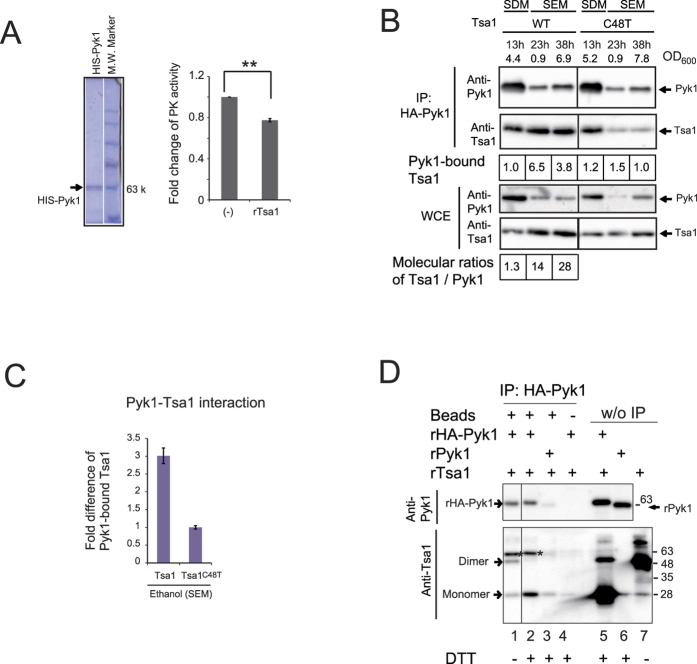
Tsa1 can bind to Pyk1 in cells during gluconeogenesis, and this interaction decreases PK activity. (**A**) The PK activity of purified HIS-tagged Pyk1 from yeast cells (approximately 0.8 μg), the quality of which is presented in the left panel (Quick-CBB staining), was repressed by the addition of recombinant Tsa1 (rTsa1, 1.0 μg). The fold changes in PK activity (N = 4) are reported, with error bars representing the standard error of the mean (right). “**” indicates p values of 0.0008. (**B**) A pull-down experiment of Tsa1 bound to HA-Pyk1. Cells expressing HA-Pyk1 and wild type Tsa1 (WT) and Tsa1^C48T^ (C48T) were grown to the exponential phase in SDM and SEM –Trp. Cells were collected at the indicated times and cell densities (OD_600_; the maximum cell density in SEM was approximately 14). Immunoprecipitates and whole cell extracts (WCE) were fractionated by SDS-PAGE with DTT in sample buffer and immunoblotting. The fold differences in the levels of Pyk1-bound Tsa1 or Tsa1^C48T^ are indicated. The molecular ratio (1.3) of Tsa1 to Pyk1 in cells growing exponentially in SDM is indicated in the data shown in Fig. 3D 32; in cells growing in SEM, this ratio was estimated by comparing the levels of Pyk1 (Tsa1) in WCE. (**C**) The fold difference in Tsa1 bound to Pyk1 was compared to that of Tsa1^C48T^. Multiple samples (N = 3) from an exponential culture (OD_600_ = 3) in SEM were analyzed as described in (**B**). (**D**) Detection of the interaction between the recombinant proteins was performed as described in the text (lanes 1 to 4). rPyk1 carrying no HA-tag was used as a negative control (lanes 3 and 6). IP was performed without anti-HA antibody beads (lane 4). Eluates (lanes 1 to 4) and the recombinant protein inputs (lanes 5 to 7; w/o IP) were subjected to SDS-PAGE and immunoblotting. Asterisks on the anti-Tsa1 antibody immunoblot (lower panel) indicate HIS-HA-Pyk1 that reacted with the anti-HIS tag antibody (see text). Treatments with DTT in SDS-PAGE sample buffer are indicated (lanes 2 to 6).

Next, we investigated the effect of Tsa1 on Pyk1 activity and the possible role of the physical and biochemical interaction between Tsa1 and Pyk1. We observed that the PK activity in tsa1/2Δ whole cell extracts grown exponentially in ethanol medium was 119% (+/− 1.19; p < 0.02) of the activity in wild type cell extracts. In addition, the PK activity of homogenously purified Pyk1 was decreased by the addition of recombinant Tsa1 (Fig. 4A). These results support the hypothesis that the PK activity of Pyk1 is downregulated in the presence of Tsa1/2, and suggest that the metabolic alterations observed in tsa1/2-deficient cells is due to higher Pyk1 activity during gluconeogenesis when grown on ethanol-containing medium.

To address the interaction between Tsa1 and Pyk1, we performed a pull-down experiment. The level of Tsa1 co-immunoprecipitated with HA-tagged Pyk1 (HA-Pyk1) was higher in cells growing exponentially in ethanol medium compared with glucose medium (Fig. 4B, the level of Tsa1 bound to Pyk1 was increased by a factor of 4∼7). Interestingly, when Tsa1 Cys48 was mutated, the Pyk1-Tsa1 interaction in cells grown in ethanol medium was significantly decreased (approximately 3-fold, p = 0.0009) (Fig. 4C). The level of Pyk1 protein considerably decreased in cells grown in ethanol medium, whereas the level of Tsa1 reciprocally increased such that the molecular ratio of Tsa1 to Pyk1 varied from 1.3 during exponential phase growth in glucose medium to more than 14 in ethanol medium (Fig. 4B, WCE). Despite the increase in the Tsa1/Pyk1 ratio, our results suggest that the direct interaction between Pyk1 and Tsa1 was enhanced by a Tsa1 Cys48-dependent mechanism when grown on ethanol-containing medium.

To determine whether the interaction of Tsa1 and Pyk1 is direct, we performed a pull-down experiment using purified recombinant proteins. rHA-Pyk1 or rPyk1 (Pyk1 without HA-tag, used as a control for a nonspecific interaction) were mixed with rTsa1. As shown in Fig. 4D, a pull-down experiment using HA-specific beads revealed that both disulfide dimers and monomers of rTsa1 were directly bound to rHA-Pyk1 (lane 1).

### Induction of the physical interaction between Tsa1 and Pyk1 in the diauxic shift in glucose medium

**Figure 5:**
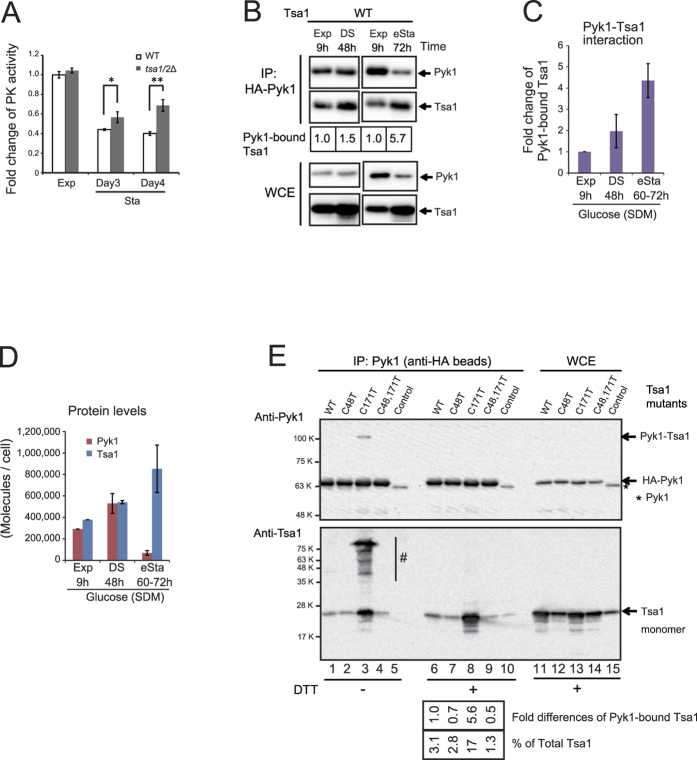
Role of Tsa1 on Pyk1 during the early stationary phase in glucose medium (SDM). (**A**) Fold changes in PK activity were determined in lysates (N = 3) that were prepared from wild type and tsa1/2∆ cells from the exponential phase (Exp) and stationary phase (Sta: after 3 days and 4 days of culture) in SDM. “*” and “**” indicate p values of 0.046 and 0.0049, respectively. (**B**) A pull-down experiment of Tsa1 bound to HA-Pyk1 in early stationary phase cells in SDM. The growth phase and culture time is indicated. A diauxic shift (DS, 48 h), and occurrence of the early stationary phase (eSta) at 72 h (see Figure Supplement 3 and below). (**C**) Fold changes in Tsa1 bound to Pyk1 in cells growing in SDM determined from four different experiments are shown. (**D**) The molecules/cell of Pyk1 and Tsa1 in cells propagating in SDM were estimated based on the data shown in Fig. 3D and quantitation of proteins in WCE from each culture. The data are the averages of four independent experiments. (**E**) Requirement of cysteine residues of Tsa1 for the interaction with Pyk1. A pull-down experiment using the indicated Tsa1 mutants bound to HA-Pyk1 as in (**B**). HA-Pyk1 was immunoprecipitated from the lysate (67 μg/lane and 111 μg/lane for HA-Pyk1 and Tsa1, respectively; lanes 1 to 10), and the whole cell extract (1 μg for Pyk1 and 0.5 μg for Tsa1; lanes 11 to 15, WCE) was separated by SDS-PAGE. BY4742 cells expressing endogenous Tsa1 and Pyk1 were used as a control. An asterisk indicates endogenous Pyk1 without a tag, which represents the nonspecific background for IP. The band intensity suggests that the IP efficiency of Pyk1 was 3.5-5.3%. The estimated levels (% of total Tsa1) and fold differences in Pyk1-bound Tsa1 are reported. Multiple slowly migrating bands (indicated as “#” in the lower panel, lane 3) were estimated at >70% of the total Tsa1.

After glucose is exhausted in the diauxic shift in SDM (medium containing glucose as a carbon source), cells begin to use overflow metabolites such as ethanol for gluconeogenesis. Therefore, we predicted that the interaction between Pyk1 and Tsa1 would be enhanced in such glucose-exhausted conditions, resulting in the downregulation of Pyk1 activity. To test this hypothesis, we examined PK activity during the early stationary phase in glucose medium and observed a greater decrease in wild type cells than in tsa1/2Δ cells (Fig. 5A). Next, we observed that the level of Tsa1 bound to Pyk1 also increased during the early stationary phase in glucose medium (Fig. 5B), and repeated experiments indicated that this increase was approximately two-fold in the diauxic shift (48 h) and four-fold in the early stationary phase (Fig. 5C, 60–72 h, 3 days). As observed in cells grown in ethanol medium, we observed a significant shift in the ratio of the levels between Tsa1 and Pyk1, demonstrating an alteration from 1.3 in the exponential and diauxic shift to more than 12 in early stationary phase (72 h) (Fig. 5D).

To examine the role of Tsa1 cysteines in the interactions between Pyk1 and Tsa1, we expressed HA-Pyk1 as well as cysteine mutants of Tsa1 in tsa1/2Δ cells and performed co-IP experiments (Fig. 5E). Mutation of Tsa1 Cys48 decreased the interaction with Pyk1 (compare lane 6 with lanes 7 and 9 in the lower panel), whereas mutation of the resolving cysteine (C171T) substantially enhanced the interaction with Pyk1 (lane 8). It is likely that Tsa1 Cys48-sulfenic acid is relatively stable after the loss of the resolving Cys (Tsa1^C171T^) and that this form of Tsa1 can preferentially attack the Cys of other proteins. The level of a slowly migrating complex of Pyk1 (indicated as Pyk1-Tsa1, 110 K; upper panel, lane 3), which is presumably a Tsa1^C171T^-HA-Pyk1 disulfide-bonded complex, was much less than the level of monomer Pyk1 (indicated as HA-Pyk1). Thus, the slowly migrating complexes of Tsa1 (lane 3 of lower panel, “#”) may consist mainly of mixed disulfide complexes of non-Pyk1 proteins linked with Tsa1 that can physically interact with HA-Pyk1.

### Requirement of Tsa1 Cys48 for the H_2_O_2_-induced interaction with Pyk1

**Figure 6:**
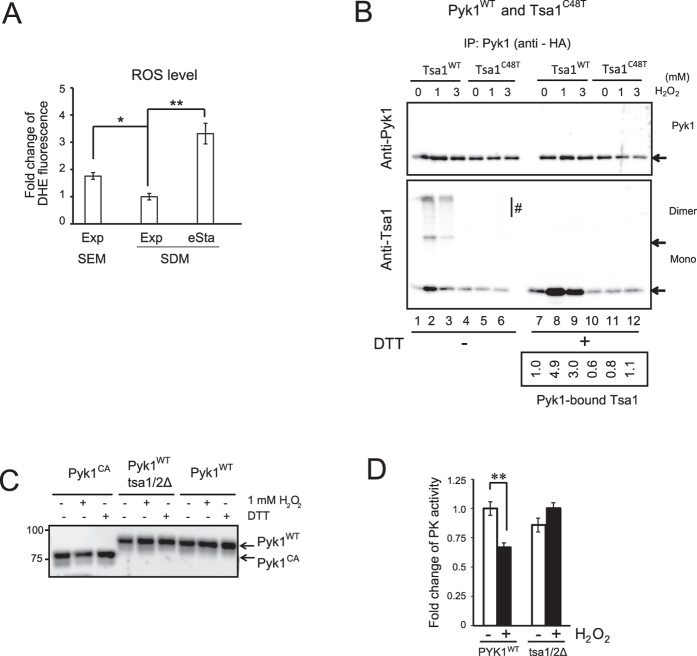
Physical interactions between Pyk1 and Tsa1 are enhanced in response to H_2_O_2_, and Cys48 of Tsa1 is required for the interaction. (**A**) ROS levels in wild type yeast cells were determined by dihydroethidium (DHE) in cells in exponential culture (OD_600_ = 1) in SEM, and in exponential (OD_600_ = 1) and early stationary phase (eSta, 3 days) cultures in SDM. “*” and “**” indicate p values of 0.005 and 0.002, respectively. (**B**) pyk1∆ tsa1/2∆ cells carrying the indicated expression plasmids were cultured for 3 days (early stationary phase) and treated with 1 or 3 mM H_2_O_2_ for 10 min. IP using HA-beads and immunoblotting with anti-Pyk1 or anti-Tsa1 antibodies were performed as described in the legend to Fig. 5. Fold differences in Pyk1-bound Tsa1 are reported. Multiple slowly migrating bands (indicated as “#”) were observed under non-reducing conditions (see the legend to Fig. 5). (**C**) The redox status of Pyk1^WT^ and Pyk1^CA^ in yeast lysates after treatment with H_2_O_2_ or DTT. Lysates were prepared from early stationary phase cells (3 days), and the lysates (10 μg) were treated with 1 mM H_2_O_2_ or 50 mM DTT for several minutes. PEGylated maleimide assays were performed as previously described^4^,^18^ using 5 mM PEGylated maleimide (MW 2 kDa). Pyk1 migrates at 65 kDa (see Fig. 4), whereas Pyk1 with seven attached PEGylated maleimide molecules migrates at 90 kDa. The difference in mobility between Pyk1^WT^ and Pyk1^CA^ is due to the C121A/C174A substitution. Pyk1^CA^ is used as a control for Pyk1 with two oxidized cysteines. (**D**) PK activity was determined in the lysate prepared from PYK1^WT^ and tsa1/2Δ cells. Lysates were prepared from early stationary phase cells (3 days) and treated with 1 mM H_2_O_2_ for a few minutes, and then the PK activity was determined. “**” indicates p values of 0.0085. The data are expressed as the mean +/− the standard error of the mean (N = 3).

The result demonstrating that the H_2_O_2_-reactive cysteine of Tsa1, Cys48, is required for an efficient interaction with Pyk1 in cells growing in ethanol medium, implies a role of metabolically produced H_2_O_2_ in the Pyk1-Tsa1 interaction. In fact, the ROS levels in cells growing exponentially in ethanol medium were significantly higher than those growing in glucose medium, and a further increase in ROS levels was observed in early stationary phase in glucose medium (Fig. 6A). The correlation between cellular ROS levels and the Pyk1-Tsa1 interaction and the requirement of Tsa1 Cys48 for an efficient interaction between Tsa1 and Pyk1 suggests that the oxidation of Cys48 by H_2_O_2_ might trigger the interaction between Pyk1 and Tsa1. Therefore, we next examined H_2_O_2_-dependent interactions and the role of the identified cysteine residues on such interactions. Stationary phase yeast cells are extremely resistant to H_2_O_2_^19^. In fact, the cells from early stationary phase were resistant to a 15-min treatment with 1 mM H_2_O_2_, but they were slightly sensitive to 3 mM and 10 mM H_2_O_2_ (Figure Supplement 4). Thus, we used these condition for the Pyk1-Tsa1 interaction. As shown in Fig. 6B, a dramatic increase in the interaction between Pyk1 and Tsa1^WT^ occurred in early stationary phase cells in response to 1 mM and 3 mM H_2_O_2_ (compare lane 7 with lanes 8 and 9 in the lower panel). The maximum interaction was increased by a factor of 5 (1 mM H_2_O_2_; lower panel, lane 8) and was equivalent to the level of interaction between Tsa1^C171T^ and Pyk1 under unstressed conditions (Fig. 5E, lower panel, lane 8). The interaction between Pyk1 and slowly migrating bands containing Tsa1 (>100 K) as well as monomer and disulfide-linked dimer Tsa1 were also enhanced by H_2_O_2_ (Fig. 6B, lower panel, lanes 2 and 3). As predicted, the interaction between Tsa1 and Pyk1 under the steady state and the H_2_O_2_-induced conditions were considerably decreased when Cys48 in Tsa1 was mutated (lower panel, lanes 10, 11 and 12), confirming the critical role of Tsa1 Cys48 in the physical interaction between Pyk1 and Tsa1. The slowly migrating complexes of Tsa1 (lower panel, lanes 2 and 3), which were only present in the anti-Tsa1 blot but not the anti-Pyk1 blot, may consist of mixed disulfide complexes of non-Pyk1 proteins linked to Tsa1 that can physically interact with HA-Pyk1.

It is possible that Pyk1 Cys reacts with Tsa1-Cys48-SOH to form a transient disulfide bond, followed by the formation of a disulfide bond within Pyk1 by a disulfide bond exchange reaction. To evaluate this possibility, we performed a PEG-maleimide assay in which the number of oxidized cysteine residues can be estimated by mobility analysis via SDS-PAGE^4^. As shown in Fig. 6C, the oxidation status of the cysteine residues of Pyk1^WT^ was not altered in response to 1 mM H_2_O_2_, despite the repression of PK activity in the lysate by H_2_O_2_ treatment (Fig. 6D). Pyk1 oxidation status was also not affected by the loss of Tsa1/2. In addition, Pyk1 protein levels were not affected by the loss of Tsa1/2. Accordingly, it is possible that the redox status of Pyk1 was unaffected by the H_2_O_2_-induced Pyk1-Tsa1 interaction, and the disulfide bond between Pyk1 and Tsa1 was only observed in the context of the Tsa1 C171T mutation (see Fig. 5E). Taken together, these results depict a unique model in which Tsa1 can downregulate Pyk1 activity due to the direct physical interaction between Tsa1 and Pyk1. While a low level of Tsa1-Pyk1 interaction is constitutive, this interaction is largely induced in response to H_2_O_2_ via a Tsa1 Cys48-dependent mechanism.

### Role of Tsa1-Cys48 oxidation on the interaction with Pyk1

**Figure 7:**
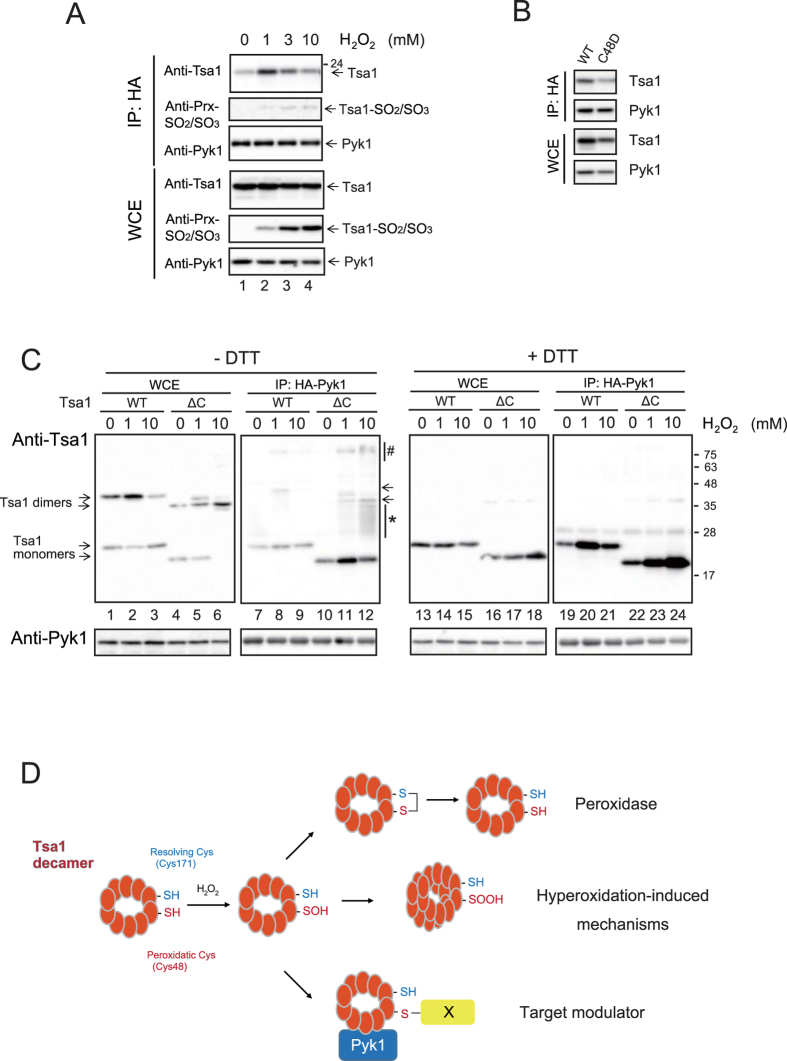
Oxidation status of Tsa1 Cys48 required for the H_2_O_2_-induced interaction with Pyk1 in early stationary phase cells. (**A**) The H_2_O_2_ concentration-dependent interaction and hyperoxidation of Cys48 were examined. pyk1∆ cells carrying an expression vector for HA-Pyk1 were cultured to early stationary phase (3 days) in SD and treated with 1 or 3 or 10 mM H_2_O_2_ for 10 min. Cell lysate preparation and IP were performed as described above. IP fractions and WCE were treated with DTT and immunoblotted using anti-Tsa1, anti-Prx-SO_2_/SO_3_ and anti-Pyk1 antibodies. The anti-Prx-SO_2_/SO_3_ antibody reacts with sulfinic acid and sulfonic acid of Tsa1-Cys48. The positions of Tsa1, Tsa1-SO_2_/SO_3_ and Pyk1 are indicated by arrows on the right side of the panels. (**B**) The interaction of Pyk1 with Tsa1^C48D^ was examined. (**C**) Tsa1/2∆ pyk1∆ cells carrying expression vectors for HA-Pyk1 and Tsa1 or a hyperoxidation-resistant mutant of Tsa1 (Tsa1∆C; ∆C) were treated with or without H_2_O_2_ (the concentration is indicated in the figure) for 10 min. WCE (lanes 1 to 6 and lanes 13 to 18) and IP fractions (lanes 7 to 12 and lanes 19 to 24) were treated with (lanes 13 to 24) or without (lanes 1 to 11) DTT, and immunoblotted using anti-Tsa1 and anti-Pyk1 antibodies. (**D**) The three independent functions of Tsa1, namely, thioredoxin (Trx)-dependent peroxidase, hyperoxidation-induced functions (such as in molecular chaperones) and redox-induced modulator of Pyk1, are presented diagrammatically. Formation of mixed disulfide bond with unknown ‘X’ proteins enhanced the interaction of Tsa1 with Pyk1.

Peroxiredoxin (Tsa1) forms dimers and decamers under physiological conditions^2^. The decamer and higher multimeric forms are induced under oxidative stress or heat stress via high H_2_O_2_-induced hyperoxidation of the peroxidatic cysteine (Cys48-thiol) to Cys48-sulfinic acid (-SOOH, hyperoxidation)^20^. Interestingly, multimerization of Tsa1 is associated with a peroxidase-to-chaperone functional switch, which decreases the levels of proteins that are aggregated by heat stress, and this phenomenon is required for heat shock resistance^20^. To examine whether the Pyk1-Tsa1 interaction depends on the H_2_O_2_ concentration and hyperoxidation of Tsa1, we treated early stationary phase yeast cells with a higher but tolerable concentration of H_2_O_2_ (see above; 1 mM, 3 mM or 10 mM) for 5 min. As shown in Fig. 7A, a significant level of Tsa1 was bound to Pyk1 in 1 mM H_2_O_2_ (lane 2, IP, anti-Tsa1), but the binding was inhibited in 3 mM and 10 mM H_2_O_2_ (lanes 3 and 4 of IP, anti-Tsa1). The Pyk1-bound Tsa1 was unreactive to anti-Prx-SO_2_/SO_3_ antibody (lanes 2 to 4, IP, anti-Prx-SO_2_/SO_3_). In contrast, the hyperoxidation level of Tsa1 Cys48 in whole cell extracts was increased in 3 mM and 10 mM H_2_O_2_ (lanes 3 and 4, WCE, anti-Prx-SO_2_/SO_3_). Thus, it is possible that the hyperoxidation of Tsa1 was not responsible for the binding to Pyk1. Furthermore, the Tsa1 C48D mutation, which can mimic hyperoxidation and enhance chaperone activity^21^, failed to enhance the binding of Tsa1 to Pyk1 (Fig. 7B).

To further elucidate the significance of sulfenic acid formation of Tsa1-Cys48, we deleted the carboxy-terminal YF-motif 2 to generate a hyperoxidation-resistant mutant of Tsa1 (Tsa1∆C). In fact, the level of hyperoxidation was decreased by the deletion mutation of Tsa1 (Figure Supplement 4B). Interestingly, in response to 1 mM H_2_O_2_ and especially to 10 mM H_2_O_2_, the Tsa1∆C efficiently formed complexes of proteins with a variety of molecular sizes of approximately 25-34 kDa and 100 kDa in the IP fraction of HA-Pyk1 (Fig. 7C, lanes 11 and 12; indicated as an asterisk and “#”, respectively). These Tsa1∆C complexes were sensitive to DTT treatment (compare lanes 11 and 12 with lanes 23 and 24, respectively). To explore the correlation between the Tsa1-Pyk1 interaction and yeast growth in SEM –Trp, we examined whether Tsa1∆C could suppress the growth phenotype. We found that the growth of cells expressing Tsa1∆C on SEM –Trp was similar to that of cells expressing Tsa1^C171T^ (Figure Supplement 4C). Thus, the enhanced interaction between Tsa1 and Pyk1 correlated to the Trp-requirement phenotype.

These results suggest that the H_2_O_2_-induced efficient interaction between Tsa1 and Pyk1 requires sulfenic acid (-SOH) formation of Tsa1-Cys48 but not hyperoxidation of Tsa1-Cys48. In addition to the observation that Pyk1-bound Tsa1^C171T^ efficiently formed mixed-disulfide complexes (see Fig. 5E), the above results suggested that the Tsa1-Cys48-SOH may induce mixed-disulfide formation with various proteins to enhance the direct physical interaction between Tsa1 and Pyk1 in a non-redox manner. Tsa1 functions as a peroxidase at low levels of H_2_O_2_ and as a hyperoxidation-induced molecular chaperone at high levels of H_2_O_2_. Our results suggested an alternative function of Tsa1 as a redox-dependent target modulator (Fig. 7D).

### Requirement of Cys48 but not the peroxidase activity of Tsa1 for efficient growth during the diauxic shift and biomass in glucose medium

**Figure 8:**
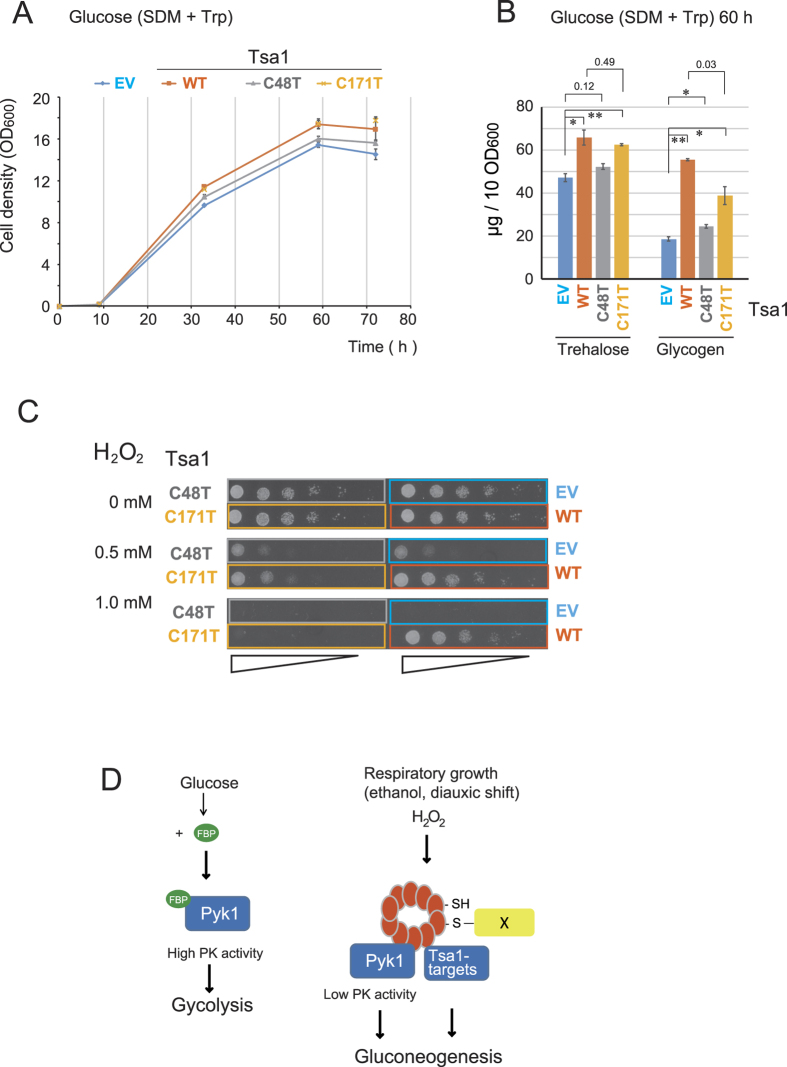
Suppression of tsa1/2Δ cell growth retardation and decrease in trehalose/glycogen during the diauxic shift by Tsa1^C171T^ (Tsa1-Cys48). (**A**) Growth curve of tsa1/2Δ cells carrying pRS315 empty vector (EV) or pRS315-TSA1 (WT) or pRS315-TSA1^C48T^ (C48T) or pRS315-TSA1^C171T^ (C171T). The cells were cultured in SDM –Leu. (**B**) The levels of trehalose and glycogen in these Tsa1 mutant-expressing cells. The cells from the above cultures were collected (10 OD_600_ units) at 36 h, and trehalose and glycogen levels were evaluated as previously described^33^. The data are expressed as the mean +/− the standard error of the mean (N = 3). “*” and “**” indicate p values < 0.02 and <0.01, respectively. Some p values greater than 0.03 are also indicated. (**C**) The above cells were spotted on SDM–Leu ager containing the indicated concentration of H_2_O_2_, and incubated for two days. (**D**) Model for Tsa1 as a H_2_O_2_ receptor and target modulator. Tsa1-Cys48 in the Tsa1 tetramer is oxidized to sulfenic acid and forms mixed disulfide followed by interactions with Pyk1. Pyk1 activity is suppressed in the absence of the allosteric activator FBP. It is possible that an unknown Tsa1 target(s) may be similarly regulated in a Tsa1-dependent manner to ensure gluconeogenesis.

Tsa1 is required for efficient growth during the diauxic shift in rich glucose medium^5^,^7^,^8^,^9^ (see Fig. 1B). Because *PYK1* is essential for vegetative growth in glucose medium, it is not possible to perform genetic analyses to estimate the role of Pyk1 in phenotype. Therefore, we examined the importance of the peroxidatic cysteine (Cys48) on phenotype in glucose medium. As shown in Fig. 8A, the growth retardation due to Tsa1/2 loss during the diauxic shift was almost completely suppressed by expressing Tsa1^C171T^; however, this did not occur in response to Tsa1^C48T^ expression. Trehalose and glycogen biosynthesis are induced during the diauxic shift via gluconeogenesis^17^. The levels of trehalose and glycogen were decreased by the loss of Tsa1/2; however, these levels were recovered by the expression of Tsa1^WT^ and Tsa1^C171T^ but not by Tsa1^C48T^ (Fig. 8B). These results suggested that Tsa1 Cys48 is essential for the regulation of the gluconeogenic flux during the diauxic shift and yeast growth. As expected^1^,^2^, yeast cells carrying either Tsa1^C48T^ or Tsa1^C171T^ were sensitive to H_2_O_2_ (Fig. 8C). Collectively, these data provide evidence for a role of the nonperoxidase function of Tsa1 in gluconeogenesis.

## Discussion

Using ethanol medium as a diauxic shift-mimicking condition, we assumed an important role for Tsa1 Cys48 and identified Pyk1 as a regulatory target for Tsa1. Because the loss of Tsa1/2 did not affect growth in ethanol medium when Trp and Phe were supplied (Fig. 1C), Tsa1 might control not only Pyk1 but also additional Tsa1 target protein(s) for efficient growth in glucose medium during the diauxic shift to ensure maximum biomass. Nevertheless, the improved understanding of the Tsa1-dependent regulatory mechanism of Pyk1 provides evidence for an alternative role of the peroxidatic cysteine Cys48 of Tsa1 in efficient gluconeogenesis. This finding is further supported by the ability of Tsa1^C171T^ expression to almost completely recover the growth retardation and to almost restore the levels of trehalose/glycogen during the diauxic shift in glucose medium (Fig. 8).

We demonstrate that after the diauxic sift, a significant level of Tsa1 is associated with Pyk1, with eight molecules of Tsa1^C171T^ estimated to be associated with each tetramer of Pyk1 (Fig. 5E) if all of the Pyk1 is completely associated. This association corresponds to the Tsa1-Pyk1 binding in cells growing in ethanol medium (Fig. 4B) as well as to the early stationary phase in response to H_2_O_2_ (Fig. 6B). The interaction results in the downregulation of PK activity in cells during gluconeogenesis. Our metabolomic analysis indicated that the level of FBP, an allosteric activator in cells in ethanol medium and cells in the early stationary phase in glucose medium (gluconeogenesis), was 4% or 5% of that in cells growing exponentially in glucose medium (glycolysis, see Table Supplement 1). Pyk1 is catalytically active, even in the presence of such a low level of FPB^10^,^13^. In addition, Pyk1 plays an important role in the synthesis of several amino acids from pyruvate via gluconeogenesis using ethanol as a carbon source^14^. Thus, we speculate that Pyk1 must be moderately downregulated to avoid energy-wasting cycling reactions and to achieve efficient gluconeogenesis. The results presented herein provide a mechanistic understanding of Pyk1 modulation under these circumstances. We show that downregulation of the PK activity of Pyk1 is governed by the physical interaction between Pyk1 and Tsa1 and that this downregulation is induced by two mechanisms. First, while the Pyk1 protein level is constitutively high from the exponential phase to the diauxic shift (48 h), it decreases to approximately 13% in the early stationary phase (post-diauxic shift; see Fig. 5D). Second, we demonstrate that the peroxidatic cysteine (Cys48) of Tsa1 plays an important role in the ROS-induced physical interaction of Tsa1-Pyk1. The H_2_O_2_-reactive catalytic center of Tsa1-Cys48 is required for proper metabolic flow (Fig. 3B) and for Tsa1 binding to Pyk1 in ethanol medium (Fig. 4B). The correlation between intracellular ROS levels (Fig. 6A) and the level of Tsa1 bound to Pyk1 in ethanol medium is consistent with the idea that ROS (H_2_O_2_)-induced oxidation of Tsa1-Cys48 is responsible for the physical interaction of Pyk1-Tsa1. In the early stationary phase in glucose medium, Tsa1-Cys48 is also essential to ensure a sufficient Pyk1-Tsa1 interaction, especially in response to H_2_O_2_ (Fig. 6B). The slowly migrating Tsa1-containing bands found in the Pyk1-IP fraction (Fig. 6B) contained proteins that could form a disulfide bond with Tsa1 in response to H_2_O_2_. Formation of the mixed disulfide bond between Tsa1-Cys48 with other proteins may induce conformational alterations in the Tsa1 tetramer to trigger physical interactions between Tsa1 and Pyk1 (Fig. 8D). Yeast cells need to adapt to changes in the availability of carbon sources and oxygen, which can result in mitochondrial ROS generation^22^. The mammalian enzyme PKM2 is regulated by the inhibition of tetramer (active form) formation mediated by several mechanisms^23^, such as serine availability, PEP levels, and tyrosine phosphorylation. However, this is not the case with Pyk1 since it is constitutively present as a tetramer^12^. Moderate changes in Pyk1 activity in the presence of low levels of the allosteric activator FBP (under low glucose) are likely to be crucial for controlling gluconeogenesis, and metabolites from PEP in yeast cells. Our results suggest that Tsa1 can act as a redox sensor and a metabolic modulator during this adaptive program: the glycolysis-to-gluconeogenesis switch (Fig. 8D).

Additional factors might also be regulated similarly by Tsa1-Cys48-dependent mechanisms to enhance trehalose/glycogen levels during the diauxic shift in glucose medium (Fig. 8). The Tsa1-interacting protein Ssb1/Ssb2 (cytosolic Hsc70/Hsp70 family) and Frd1 are additional candidate Tsa1 target proteins that may be correlated to phenotype. Hsc70 is a multi-functional chaperone that is present at a level comparable to Tsa1. Recently, it has been shown that the lifespan is extended by the enhanced physical interaction between Tsa1 and Hsp70^24^. This interaction is induced by hyperoxidation of Tsa1 by H_2_O_2_, and enhances the recovery of aggregated proteins during aging. In addition, Frd1 is a cytosolic soluble fumarate reductase^25^ that may affect gluconeogenesis. We examined whether Frd1 could affect gluconeogenesis. However, we found that neither the disruption of *FRD1* nor the overexpression of Frd1 affected the observed phenotype (our unpublished observation). Thus, the identification of additional Tsa1 target(s) is necessary to complete our understanding of the role of Tsa1-Cys48 in gluconeogenesis.

Tsa1 is a major yeast Prx that performs not only thioredoxin-dependent peroxidase activities but also chaperone functions^20^, both of which are conserved in mammalian systems^26^. Our results suggest a novel function of Prx that involves redox regulation of the peroxidatic cysteine. Consequently, this is the first report to demonstrate that Tsa1 has a dual function as an H_2_O_2_ receptor and as a modulator of target proteins by direct physical interactions.

## Methods

### Yeast media and culture

Yeast cells were grown at 30 °C in synthetic medium (yeast nitrogen base without amino acids, BD Difco, NY, USA) supplemented with 2% glucose (SDM) or 2% ethanol (SEM) and with the following amino acid and nucleotide supplements^27^: 40 μg/ml adenine hemisulfate, 20 μg/ml L-arginine monohydrochloride, 100 μg/ml L-aspartic acid, 100 μg/ml L-glutamic acid monosodium salt, 68 μg/ml L-lysine monohydrochloride, 20 μg/ml L-methionine, 50 μg/ml L-phenylalanine, 375 μg/ml L-serine, 200 μg/ml L-threonine, 40 μg/ml L-tryptophan, 30 μg/ml L-tyrosine, 150 μg/ml L-valine, 20 μg/ml L-histidine, 60 μg/ml L-leucine and 20 μg/ml uracil. We also used synthetic glucose medium without amino acid supplements but containing nutrients for auxotrophy (SD): 72 μg/ml L-lysine monohydrochloride, 80 μg/ml L-histidine, 240 μg/ml L-leucine and 80 μg/ml uracil. Yeast cells growing in the exponential phase were washed and diluted in an appropriate medium to a cell density of 0.1 OD_600_ and cultured in a rotary shaker at 30 °C. The ROS level was determined as previously described^5^.

Spot assays were performed as follows: a series of one-fifth dilutions of 5 × 10^4^ of the appropriate cells prepared under the indicated conditions were spotted on an agar plate (2% Bacto agar, BD Difco), and the agar plate was incubated at 30 °C for 2 days (SDM), 3 days (SDM containing H_2_O_2_), or 4–5 days (SEM). Amino acids and unspecified reagents were purchased from Nacalai Tesque, Kyoto, Japan.

### Yeast metabolomics analysis

Yeast metabolites were extracted from cell cultures (10 OD_600_ units) as previously described^28^, and metabolomic analysis was performed using capillary electrophoresis mass spectrometry^29^.

### Measurements of intracellular ROS by flow cytometry

Dihydroethidium (DHE; Wako pure chemical, Osaka, Japan) was dissolved in DMSO (1.9 mg/ml). Yeast cell cultures (0.5 ml x OD_600nm_ = 1) were mixed with 5 μl of DHE stock solution and incubated for 10 min at room temperature. After the cells were washed with medium, the fluorescence of each group of cells (10,000 cells) was measured with a FACSCalibur (Becton, Dickinson and Company, Franklin Lakes, NJ, USA) using FL3. We determined a threshold level for each fluorescent probe by measuring the background fluorescence of unlabeled cells and comparing the average fluorescence values, which were obtained using appropriate conditions (laser levels and detector gain) that had been determined for each fluorescent probe.

### Manipulation of genes and yeast strain construction

We manipulated a parental *S. cerevisiae* strain BY4742 (*MAT*α *his3*Δ1 l*eu2*Δ0 *lys2*Δ0 *ura3*Δ0) (EUROSCARF, Frankfurt, Germany) derived from S288C to construct the strains listed in Table S2. Tsa1 and Tsa2 are homologous Prxs that are present in the cytoplasm and have similar peroxidase activity. Although *TSA1* is the most abundant Prx (more than 90% of the total Prx 7), *TSA2* expression (approximately 1% of the total Prx) is induced in a *tsa1*Δ genetic background and tends to compensate for the lost cellular antioxidant activity^8^. Thus, we examined *tsa1*Δ*tsa2*Δ double knockout cells (abbreviated hereafter as *tsa1/2*Δ and tsa1/2Δ for genotype and strain, respectively). The parental strain BY4742 was used as wild type cells.

To construct *pyk1*::kanMX, we replaced a coding region of *PYK1* with the kanMX gene from pUG6^30^. These DNA fragments first form as plasmid DNA and are then PCR-amplified. After transfection of the yeast cells, appropriate transformants were selected on agar plates containing G418 (kanMX) and hygromycin B (hphMX)^31^. To construct the Cys121/Cys174 mutant of *PYK1 (PYK1*^CA^), two codons, “TGT” at Cys121 and Cys174, were mutated to “GCT” (Ala) using a PCR-based method. Other mutations were introduced in a similar manner. We introduced DNA fragments encoding a duplicated hemagglutinin epitope HA_2_-tag (MAAMYPYDVPDYAGSYPYDVPDYAGSRV) at the 5′ end of the coding sequence of Pyk1 (pRS313-HA-PYK1). Next, we used a basic yeast gene replacement method as described elsewhere^27^. To replace the genomic allele of *PYK1* with a *PYK1* cysteine mutant, we first introduced the *URA3* gene into the *Nde*I site at –285 of the *PYK1* coding region, and the resulting *PYK1-pro::URA3* allele was replaced at the corresponding genomic allele. We then again replaced the genomic locus with a DNA fragment containing *PYK1*^WT^ or *PYK1*^CA^. We confirmed all of the genomic replacements by PCR. The list of these strains is provided in Table Supplement 2.

The *TSA1* gene (−764 to +343 from the Tsa1 ORF) was cloned into pRS315 (CEN-ARS origin, *LEU3*). Next, codon 48 “TGT” (Cys) was mutated to “GAC” (Asp). We also deleted a C-terminal region corresponding to codon 180 to 196 to prepare pRS315-TSA1ΔC. Other Tsa1 mutants (Tsa1^C48T^, Tsa1^C171T^, Tsa1^C48,171T^) have been previously described^18^.

### Affinity purification and identification of Tsa1^C171T^ binding protein

To decrease non-specific binding during the purification of the Tsa1 protein and its binding proteins from yeast cells, we replaced the polyhistidine (His)-tag region in the Tsa1 expression plasmids^18^ with a HAT-tag (histidine affinity tag; amino acid sequence “KDHLIHNVHKEHAHAHNK”) to produce pRS-315-HAT-tag-TSA1^C171T^ and pRS-315-HAT-tag-TSA1^C48T^. We treated an exponential-phase culture (0.6 OD_600_) of tsa1Δ cells carrying a pRS-315-HAT-tag-TSA1^C171T^ SDM –Leu with 0.5 mM H_2_O_2_ in fresh SDM –Leu for 5 min. The cells were washed with 10 mM *N*-ethylmaleimide (NEM) and suspended in Buffer L (50 mM NaH_2_PO_4_, pH 8.0, 300 mM NaCl, 0.2% Triton X-100, 50 mM NEM, 1 mM phenylmethanesulfonyl fluoride, 0.5 mM N-*p*-tosyl-L-phenylalanine chloromethyl ketone, 0.05 mM *N*_α_-tosyl-L-lysine chloromethyl ketone hydrochloride, 2 μg/ml pepstatin A, 5 μg/ml aprotinin and 3 μg/ml leupeptin). The cells were then frozen and disrupted in liquid nitrogen by grinding in a mortar placed on dry ice. The supernatant lysate was collected after centrifugation (15,000 × *g*, 10 min), and purification was performed using TALON® Metal Affinity Resins (Takara Clontech Laboratories Inc. CA, USA). After incubation of 1 ml of the resins with the lysate, the resins were washed two times with Buffer W1 (50 mM NaH_2_PO_4_, pH 8.0, 300 mM NaCl, 2% Triton X-100 and 10 mM imidazole), eluted with 1 ml of elution buffer (50 mM NaH_2_PO_4_, pH 8.0, 300 mM NaCl, and 150 mM imidazole), and concentrated using Vivaspin 500 devices (30,000 MWCO PES, Vivaproducts, Littleton, MA USA). The proteins were quantified with a *DC* Protein Assay kit (Bio-Rad Laboratories, Hercules, CA) with BSA as the standard. Tsa1-bound proteins (100 μg) in sample buffer (50 mM Tris-HCl, pH 6.8, 2% SDS, 0.1% bromophenol blue, and 10% glycerol) were separated by two-dimensional gel electrophoresis (non-reduced and reduced in the first and second dimensions, respectively). Polyacrylamide gel electrophoresis was conducted using SDS-PAGE (10%, with an acrylamide to bisacrylamide ratio of 29:1). We used a disk gel (3.5-mm inner diameter; 14 cm and 2 cm for the running gel and stacking gel, respectively) for the first dimension of electrophoresis (2 mA/disk) and a slab gel (1-mm thick, and 165 mm and 30 mm for the running and stacking gels, respectively) for the second dimension. After the first dimension of electrophoresis, the disk gel was soaked in a buffer containing 50 mM Tris-HCl, pH 6.8, 2% SDS, 0.1% BPB, 10% glycerol and 5% 2-mercaptoethanol for 60 min prior to the second dimension of electrophoresis. The slab gels were stained using a Silver Stain II Kit (Wako Pure Chemical) using 0.2 g/ml sodium thiosulfate as the developer. Proteins in the spots were carboxymethylated and trypsinized. The resulting peptides were analyzed by TOF-MS/MS using a MALDI-Qq-TOF MS/MS QSTAR Pulsar *i* (Applied Biosystems, ThermoFisher Scientific).

### Whole cell extract preparation, co-immunoprecipitation of Pyk1 and immunoblotting

We successfully identified the Pyk1-Tsa1 complex using co-IP experiments. pyk1∆ tsa1/2∆ cells carrying an expression vector for HA-Pyk1 and wild type Tsa1 or Tsa1 mutant were collected by centrifugation at 5,000 × *g* for 1 min and then suspended in IP buffer (100 mM 2-morpholinoethanesulfonic acid, pH 7.0, 200 mM KCl, 15 mM MgCl_2_, 5% glycerol, 0.15% NP-40, 50 mM N-ethylmaleimide (NEM)) containing 2 mM phenylmethanesulfonyl fluoride, 1 μg/ml leupeptin and 1 μg/ml pepstatin A. NEM was added to block free Cys-SH to prevent artificial thiol-disulfide exchange reactions. The cells were then frozen in liquid nitrogen and disrupted by shaking at 2,000 rpm for 30 sec with a Multi-beads shocker (Yasui Kikai Corporation, Osaka, Japan). The whole cell extract (WCE; 400 μg) was mixed with anti-HA antibody beads (Wako Pure Chemical) or with anti-HA Affinity Matrix (Roche Diagnostics, Basel, Germany) at 4 °C for 3 h. Bound proteins were eluted from the beads with 90 μl of sample buffer (50 mM Tris-HCl, pH 6.8, 2% SDS, 0.1% bromophenol blue, and 10% glycerol) with or without 50 mM DTT. The immunoprecipitates were analyzed by SDS-PAGE (10% and 15% acrylamide gel for Pyk1 and Tsa1, respectively) and immunoblotted using anti-Pyk1 rabbit polyclonal antibodies, anti-Tsa1 rabbit polyclonal antibodies^18^, and anti-Prx-SO_2_/SO_3_ antibody (LabFrontier, Seoul, Korea). Chemiluminescent images of the immunoblot were obtained using Immobilon Western Chemiluminescent HRP Substrate (Millipore, Billerica, MA, USA) and the VersaDoc™ imaging system (Bio-Rad). Anti-Pyk1 polyclonal antibody was prepared by immunizing a rabbit with bacterially expressed HIS-Pyk1 protein as previously described^4^. Quick-CBB (Wako pure chemical) was used for Coomassie brilliant blue staining.

### Determination of pyruvate kinase activity

We prepared WCE as described above except that NEM and NP40 were omitted, and then determined the pyruvate kinase (PK) activity in the WCE as previously described^12^. FBP was omitted from the reaction mixture. Briefly, 800 μM PEP was added to a reaction mixture (150 μl) containing 100 mM 2-morpholinoethanesulfonic acid, pH 7.0, 200 mM KCl, 15 mM MgCl_2_, 5% glycerol, 150 μM NADH, 2.4 mM ADP, 25 U of lactate dehydrogenase (Toyobo, Osaka, Japan) and 20 μg of WCE. NADH oxidation was determined at 30-s intervals by measuring the OD_340_ using a spectrophotometer (Varioskan Flash, Thermo Scientific, MA, USA).

### Interaction between recombinant Pyk1 and Tsa1 proteins

HA-*PYK1* and *TSA1* were cloned into pET15b (Millipore), and the recombinant proteins were isolated from *E. coli*. Recombinant HIS-HA-Pyk1 (20 μg) and recombinant HIS-Tsa1 (23 μg) were mixed in 24 mM KH_2_PO_4_/K_2_HPO_4_ (pH 7.0) and 10 mM MgSO_4_ (50 μl) with or without 10 mM DTT and then incubated for 10 min. The reaction mixtures were then mixed with IP buffer containing 50 mM NEM (600 μl) that included anti-HA antibody beads and incubated for 1 h with rotation. The beads were washed three times with IP buffer and then boiled in sample buffer with or without 50 mM DTT. HIS-HA-Pyk1 was detected using anti-HIS-Tsa1 antibody (Fig. 4D) because antibody against the HIS tag had been generated during rabbit immunization rabbit with HIS-Tsa1^4^.

### Redox status of Pyk1 in yeast cells

Lysates of yeast cells expressing HA-Pyk1 were prepared as previously described^4^,^18^ with modifications. Briefly, yeast lysates treated with H_2_O_2_ or DTT were suspended in 10% trichloroacetic acid (TCA) to terminate the thiol-disulfide exchange reactions of the cellular proteins. The lysates were prepared in TCA and washed with acetone under nitrogen. The lysate was dissolved in a degassed buffer containing 50 mM Tris-HCl, pH 8.0, 0.15 M NaCl, 1 mM EDTA, 8 M urea, a complete protease inhibitor cocktail (Roche Diagnostics, Basel, Germany) and 5 mM PEGylated maleimide (MW 2,000) (SUNBRIGHT® ME-020MA, NOF Corporation, Tokyo) for 15 min and then fractionated by SDS-PAGE.

### Reproducibility and statistical analysis

All experiments were repeated at least three times, and representative results are reported. Multiple independent replicates (at least n = 3, except as indicated) were performed for each experiment, and the data are presented as the mean of three independent experiments with the standard error of the mean. Statistical comparisons between two conditions were conducted using Student’s *t*-test.

## Additional Information

**How to cite this article**: Irokawa, H. *et al.* Redox-dependent Regulation of Gluconeogenesis by a Novel Mechanism Mediated by a Peroxidatic Cysteine of Peroxiredoxin. *Sci. Rep.* **6**, 33536; doi: 10.1038/srep33536 (2016).

## Supplementary Material

Supplementary Information
