# Supplementary material for: Redox-dependent Regulation of Gluconeogenesis by a Novel Mechanism Mediated by a Peroxidatic Cysteine of Peroxiredoxin
Source: Sci Rep. 2016 Sep 16;6:33536. doi: 10.1038/srep33536 (PMC5025857; doi:10.1038/srep33536)
Supplement: Supplementary Information [file srep33536-s1.pdf]

Supplemental information

Redox-dependent Regulation of Gluconeogenesis by a Novel Mechanism Mediated by a Peroxidatic-Cysteine of Peroxiredoxin

Hayato Irokawa, Tsuyoshi Tachibana, Toshihiko Watanabe, Yuka Matsuyama, Hozumi Motohashi, Ayako Ogasawara, Kenta Iwai, Akira Naganuma and Shusuke Kuge

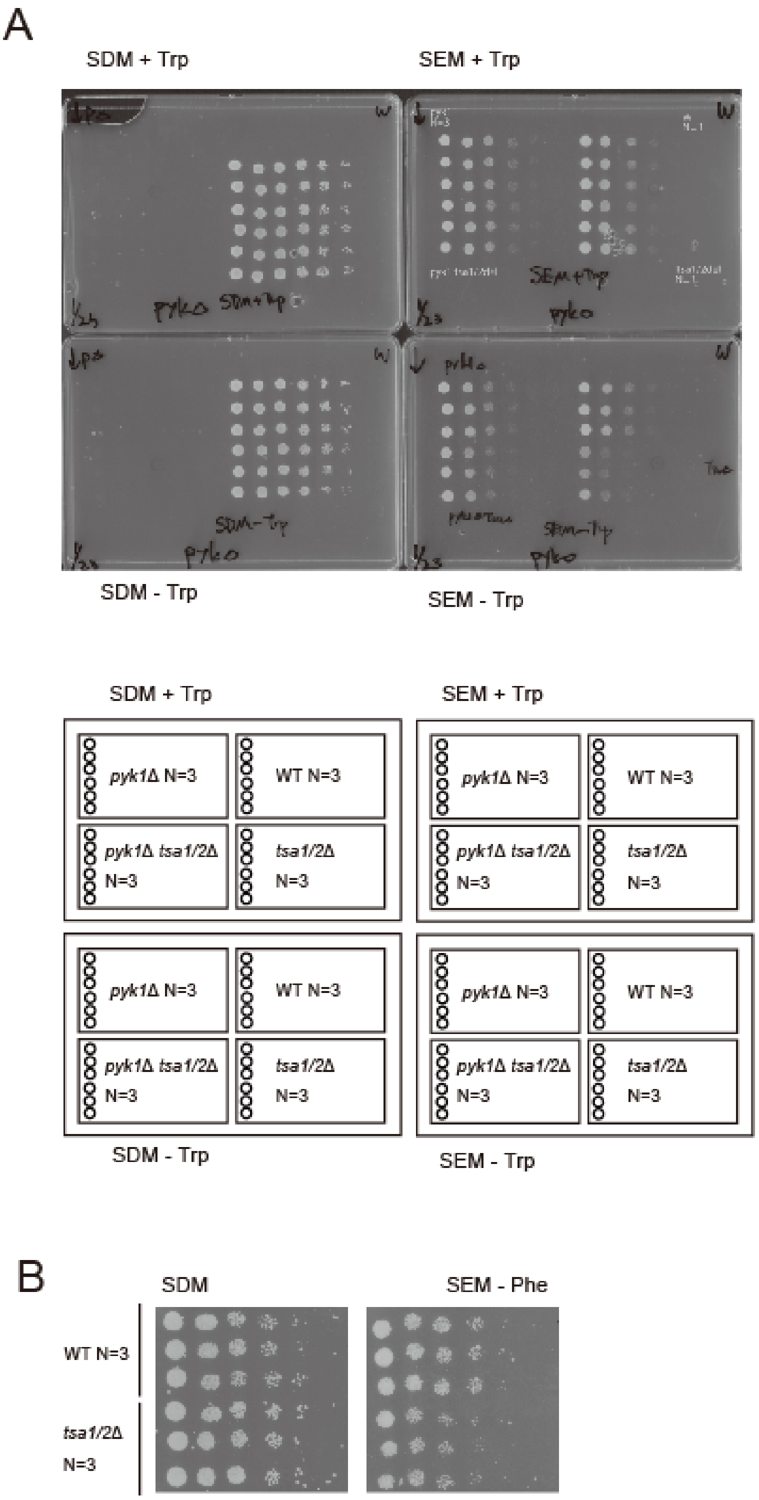

Figure supplement 1 (Related to *Figures 1D, 1E* and *3E*): Loss of Tsa1/2 leads to growth suppression in Trp- or Phe-free ethanol medium in the presence of Pyk1.

A. Original data (N = 3) for Figures 1D (upper panel) and 3E. **B**. Original data (N = 3) for Figure 1 D (lower panel). *PYK1*-disruption mutant leads to a growth defect on glucose medium[1].

A: VDE

```
                      *
   1 CFAKGTNVLM ADGSIECIEN IEVGNK VMGKDGRPREVIKL PRGRETMYSV
                               *
  51 VQKSQHRAHK SDSSREVPEL LKFTCNATHE LVVRTPRSVR RLSRTIKGVE
 101 YFEVITFEMG QKKAPDGRIV ELVKEVSKSY PISEGPERAN ELVESYRKAS
 151 NKAYFEWTIE ARDLSLLGSH VRKATYQTYA PILYENDHFF DYMQKSKFHL
                                                         *
 201 TIEGPKVLAY LLGLWIGDGL SDRATFSVDS RDTSLMERVT EYAEKLNLCA
 251 EYKDRKEPQV AKTVNLYSKV VRGNGIRNNL NTENPLWDAI VGLGFLKDGV
 301 KNIPSFLSTD NIGTRETFLA GLIDSDGYVT DEHGIK ATIKTIHTSVRDGL
 351 VSLARSLGLV VSVNAEPAKV DMNGTKHKIS YAIYMSGGDV LLNVLSKCAG
 401 SKKFRPAPAA AFARECRGFY FELQELKEDD YYGITLSDDS DHQFLLANQV
 451 VVHN
```

B: Pyk1

```
   1 MSRLERLTSL NVVAGSDLRR TSIIGTIGPK TNNPETLVAL RKAGLNIVRM
  51 NFSHGSYEYH KSVIDNARKS EELYPGRPLA IALDTKGPEI RTGTTTNDVD
 101 YPIPPNHEMI FTTDDKYAKA CDDKIMYVDY KNITKVISAG RIIYVDDGVL
 151 SFQVLEVVDD KTLKVKALNA GKICSHKGVN LPGTDVDLPA LSEKDKEDLR
 201 FGVKNGVHMV FASFIRTAND VLTIREVLGE QGKDVKIIVK IENQQGVNNF
 251 DEILKVTDGV MVARGDLGIE IPAPEVLAVQ KKLIAKSNLA GKPVICATQM
 301 LESMTYNPRP TRAEVSDVGN AILDGADCVM LSGETAKGNY PINAVTTMAE
 351 TAVIAEQAIA YLPNYDDMRN CTPKPTSTTE TSLPRVAAVF EQKAKAIIVL
 401 STSGTTPRLV SKYRPNCPII LVTRCPRAAR FSHLYRGVFP FVFEKEPVSD
 451 WTDDVEARIN FGIEKAKEFG ILKKGDTYVS IQGFKAGAGH SNTLQVSTV
```

C: Ssb1/2

```
   1 MAEGVFQGAI GIDLGTTYSC VATYESSVEI IANEQGNRVT PSFVAFTPQE
  51 RLIGDAAKNQ AALNPRNTVF DAKRLIGRRF DDESVQKDMK TWPFKVIDVD
 101 GNPVIEVQYL EETKTFSPQE ISAMVLTKMK EIAEAKIGKK VEKAVITVPA
 151 YFNDAQRQAT KDAGAISGLN VLRIINEPTA AAIAYGLGAG KSEKERHVLI
 201 FDLGGGTFDV SLLHIAGGVY TVKSTSGNTH LGGQDFDTNL LEHFKAEFKK
 251 KTGLDISDDA RALRRLRTAA ERAKRTLSSV TQTTVEVDSL FDGEDFESSL
 301 TRARFEDLNA ALFKSTLEPV EQVLKDAKIS KSQIDEVVLV GGSTRIPKVQ
 351 KLLSDFFDGK QLEKSINPDE AVAYGAAVQG AILTGQSTSD ETKDLLLLDV
 401 APLSLGVGMQ GDIFGIVVPR NTTVPTIKRR TFTTVSDNQT TVQFPVYQGE
 451 RVNCKENTLL GEFDLKNIPM MPAGEPVLEA IFEVDANGIL KVTAVEKSTG
 501 KSSNITISNA VGRLSSEEIE KMVNQAEEFK AADEAFAKKH EARQRLESYV
 551 ASIEQTVTDP VLSSKLKRGS KSKIEAALSD ALAALQIEDP SADELRKAEV
 601 GLKRVVTKAM SSR
```

D: Yol057w

```
   1 MSHFFADHDA PLSMLSVKTE YFPQLTDKEQ KYAHFMSKAS HAGSRVVMRQ
  51 VSHESEPIFD LILAIHSKLN GKYPEDDITQ KQQTGLYLEY VSQFLSNLGN
 101 FKSFGDTKFI PRCEVKFFKQ LLELAKINPC SSPLTLSPVD VNHEFTSHHL
 151 FSTINELIDI GIYHVEEKAA LLGFPSQGYT SAYYLGLPVT PEDMALLKEQ
 201 LFAELAILPE NTRINKVGEN SFQIWVASEN VKNQITETYP SGQITLSNAV
 251 TKVEFIFGDH SREMRLVASY LKEAQKFAAN DTQKAMLQEY INHFVTGSSQ
 301 AHKEAQKLWV KDISPVIETN IGFIETYREP SGIIGEFESL VAIQNKERTA
 351 KFSSLVNNAE EFISLLPWSK DYEKPIFNPP DFTSLEVLTF TGSGIPAGIN
 401 IPNYDDVRLK IGFKNVSLGN ILSAAAKSSS KHPPSFISQE DRPIFEKYQS
 451 DSFEVQVGIH ELLGHGSGKL LTEFTDGFNF DKENPPLGLD GKPVSTYYKV
                       *
 501 GETWGSKFGQ LAGPFEECRA EVIAMFLLTN KKILDIFGFH DVESQDKVIY
 551 AGYLQMARAG LLALEYWNPK TGKWGQPHMQ ARFSIMKTFM KHSTDKNFLK
                                *             *
 601 LEMNSTNDDF AIKLDKSLIK TAGHECVKDY LKHLHVYKCS GDVEQGSKYF
 651 IDRSTVTPDL ASLRDIVLSK RLPRRQFIQS NSYIDDNNKV TLKEYDETPQ
 701 GMLQSFLDRE L
```

E: FRDS1

```
   1 MSLSPVVVIG TGLAGLAAAN ELVNKYNIPV TILEKASSIG GNSIKASSGI
  51 NGACTETQRH FHIEDSPRLF EDDTIKSAK GKGVQELMAKL ANDSPLAIEW
 101 LKNEFDLKLD LLAQLGGHSV ARTHRSSGKL PPGFEIVSAL SNNLKKLAET
 151 KPELVKINLD SKVVDIHEKD GSISAVVYED KNGEKHMVSA NDVVFCSGGF
 201 GFSKEMLKEY APELVNLPTT NGQQTTGDGQ RLLQKLGADL IDMDQIQVHP
 251 TGFIDPNDRS SSWKFLAAES LRGLGGILLN PITGRRFVNE LTTRDVVTAA
         *
 301 IQKVCPQEDN RALLVMGEKM YTDLKNNLDF YMFKKLVQKL TLSQVVSEYN
 351 LPITVAQLCE ELQTYSSFTT KADPLGRTVI LNEFGSDVTP ETVVFIGEVT
 401 PVVHFTMGGA RINVKAQVIG KNDERLLKGL YAAGEVSGGV HGANRLGGSS
        *
 451 LLECVVFGRT AAESIANDRK
```

Figure supplement 2 (Related to *Figure 3C and 3D*): Isolation and identification of Tsa1 binding proteins.

Amino acid sequences for corresponding spots a-e in Figure 3C represented as A to E, respectively. Underlined regions are peptide regions that were identified in tryptic digests by peptide mass fingerprinting. Cys residues in the identified peptide are indicated as '*'. None of the Cys residues were modified by NEM, although these Cys residues were carboxymethylated by treatment during trypsinization.

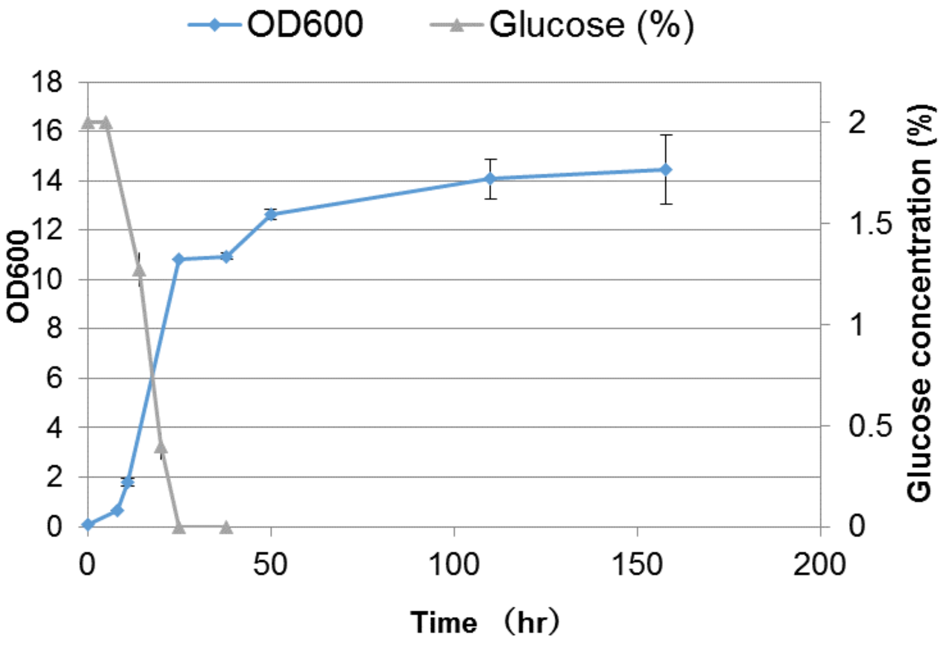

Figure supplement 3 (Related to *Figure 5B*): Wild type yeast growth and consumption of glucose in glucose medium

The growth curve and glucose consumption of wild type yeast ($PYK1^{WT}$) in SD medium. Glucose consumption was evaluated as described previously[2].

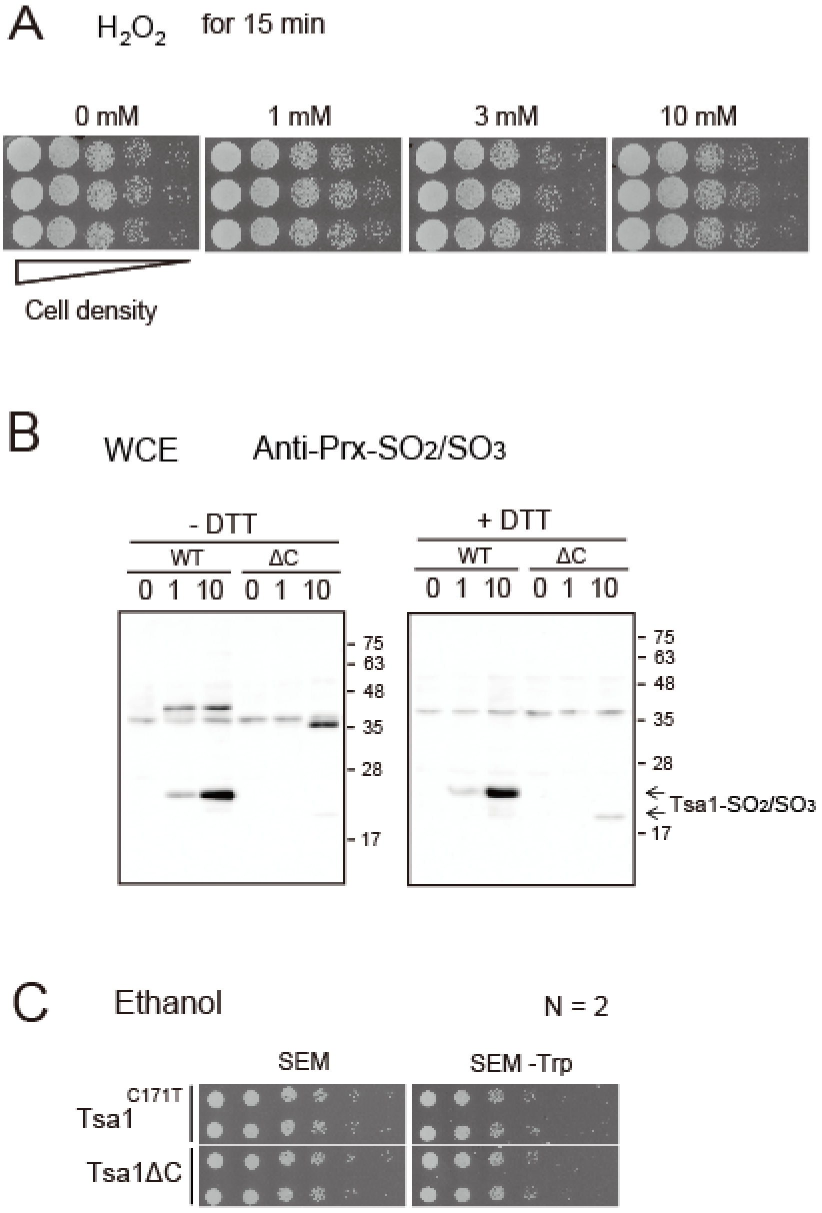

Figure supplement 4 (Related to *Figure 7*):Characterization of the hyper oxidation resistant mutant Tsa1ΔC.

A. Resistance of Yeast cells to H_2O_2. Wild type cells ($PYK1^{WT}$, N = 3) were cultured for 3 days. The cells were diluted to OD_{600} = 1 and treated with 1 mM or 10 mM or 3 mM H_2O_2 for 15 min. Then, a series of one-fifth dilutions of the cells were spotted on YPAD agar (1% Bacto peptone, 0.5% Bacto yeast extract, 2% glucose, 40 µg/ml adenine hemisulfate, 2% agar). The plates were incubated at 30°C. **B.** Whole cell Lysates described in Figure 7C were immunoblotted by anti-Prx-SO_2/SO_3 antibody. The positions Tsa1-SO_2/SO_3 and Tsa1ΔC-SO_2/SO_3 are indicated by arrows on the right side of the panels. **C**. Spot assay for tsa1/2Δ cells carrying plasmids $Tsa1^{C171T}$ and Tsa1Δ on SEM or SEM –Trp (N = 2). For plasmid selection, Leu was also omitted from the medium.

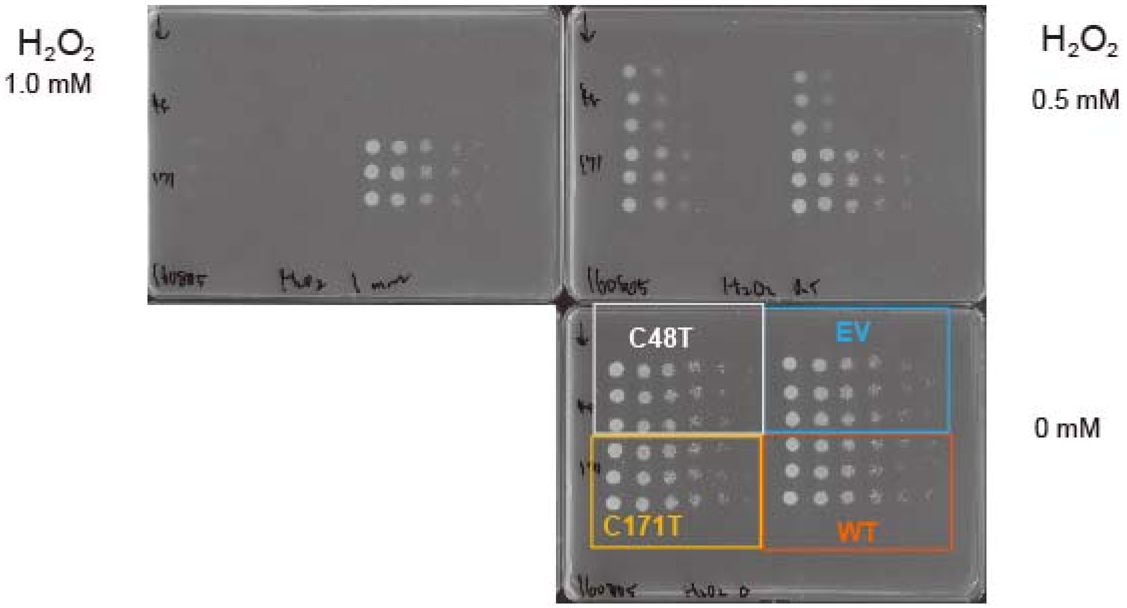

Figure supplement 5 (Related to *Figure 8*): Original image of the spot assay in Figure 8B (N = 3).

Table supplement 1 (Related to *Discussion*)

FBP (pmol / 10 OD_{600})

	Average (n = 3)	Standard error of the mean
Exponential (Glucose medium)	7626	667
Early stationary (Glucose medium)	336	17
Exponential (Ethanol medium)	425	5

FBP concentration in exponential phase cells and early stationary phase cells in SDM (glucose medium), and exponential phase cells (adapted from ***Figure 2B***) in SEM –Trp (ethanol medium).

Table supplement 2 – *Saccharomyces cerevisiae* strains used in this study (related to *Materials and Methods*)

BY4742	*MATα his3Δ1 lys2Δ0 ura3Δ0*	Euroscarf
tsa1Δ	*tsa1*::kanMX (from BY4742)	Tachibana et al. 2009
tsa1/2Δ	*tsa1*::hphMX *tsa2*::kanMX (from tsa1Δ)	Watanabe et al. 2014
pyk1Δ	*pyk1*::kanMX (from BY4742)	this study
PYKl pro::URA3	*PYK1*::*URA3* (from BY4742)	this study
PYKl pro::URA3 tsa1/2Δ	*PYK1*::*URA3* (from tsa1/2Δ)	this study
$PYK1^{WT}$	$\mathit{PYK1}^{WT}$ (from PYKl pro::URA3)	this study
$PYK1^{CA}$	$\mathit{PYK1}^{CA}$ (from PYKl pro::URA3)	this study
$PYK1^{WT}$ tsa1/2Δ	$\mathit{PYK1}^{WT}$ (from PYKl pro::URA3Δ tsa1/2Δ)	this study
tsa1/2Δ URA3	*tsa1*::hphMX *tsa2*::Ca*URA3* (from tsa1Δ)	Watanabe et al. 2014
pyk1Δ tsa1/2Δ	*pyk1*:: kanMX *tsa1*::hphMX *tsa2*::Ca*URA3* (from tsa1/2Δ URA3)	this study
pyk1Δ	*pyk1*:: kanMX (from BY4742)	this study

Supplementary References

1 Sprague, G. F., Jr. Isolation and characterization of a Saccharomyces cerevisiae mutant deficient in pyruvate kinase activity. *J Bacteriol* **130**, 232-241 (1977).

2 Watanabe, T., Irokawa, H., Ogasawara, A., Iwai, K. & Kuge, S. Requirement of peroxiredoxin on the stationary phase of yeast cell growth. *J Toxicol Sci* **39**, 51-58, doi:DN/JST.JSTAGE/jts/39.51 [pii] (2014).
